# Optimisation of Simultaneous Saccharification and Fermentation (SSF) for Biobutanol Production Using Pretreated Oil Palm Empty Fruit Bunch

**DOI:** 10.3390/molecules23081944

**Published:** 2018-08-03

**Authors:** Nur Atheera Aiza Md Razali, Mohamad Faizal Ibrahim, Ezyana Kamal Bahrin, Suraini Abd-Aziz

**Affiliations:** Department of Bioprocess Technology, Faculty of Biotechnology and Biomolecular Sciences, Universiti Putra Malaysia, 43400 UPM Serdang, Selangor, Malaysia; atheera.aiza@gmail.com (N.A.A.M.R.); faizal_ibrahim@upm.edu.my (M.F.I.); ezyana@upm.edu.my (E.K.B.)

**Keywords:** simultaneous saccharification and fermentation (SSF), oil palm empty fruit bunch (OPEFB), biobutanol, *Clostridium acetobutylicum* ATCC 824, optimisation

## Abstract

This study was conducted in order to optimise simultaneous saccharification and fermentation (SSF) for biobutanol production from a pretreated oil palm empty fruit bunch (OPEFB) by *Clostridium acetobutylicum* ATCC 824. Temperature, initial pH, cellulase loading and substrate concentration were screened using one factor at a time (OFAT) and further statistically optimised by central composite design (CCD) using the response surface methodology (RSM) approach. Approximately 2.47 g/L of biobutanol concentration and 0.10 g/g of biobutanol yield were obtained after being screened through OFAT with 29.55% increment (1.42 fold). The optimised conditions for SSF after CCD were: temperature of 35 °C, initial pH of 5.5, cellulase loading of 15 FPU/g-substrate and substrate concentration of 5% (*w*/*v*). This optimisation study resulted in 55.95% increment (2.14 fold) of biobutanol concentration equivalent to 3.97 g/L and biobutanol yield of 0.16 g/g. The model and optimisation design obtained from this study are important for further improvement of biobutanol production, especially in consolidated bioprocessing technology.

## 1. Introduction

Recently, gasoline consumption has been increased tremendously due to the increase in human population that leads to an increase in energy consumption. Intense gasoline consumption may leads to an energy crisis and environmental pollution [1,2]. Therefore, current interest has shifted towards greener biofuels such as biobutanol, bioethanol, biodiesel, biohydrogen and biomethane. Among these biofuels, biobutanol possesses attractive characteristic as a replacement fuel for gasoline or as a fuel additive [3]. Biobutanol produced from the biological route of acetone-butanol-ethanol (ABE) fermentation by *Clostridia* species has the same chemical properties with butanol produced from petrochemical route. This biobutanol offers an alternative solution for energy security and mitigates greenhouse gases emission as it can be derived from sustainable and renewable substrates [4,5]. Biobutanol has a good blending ability with gasoline at any ratio or as a drop in an existing vehicle engine. Besides, biobutanol has a comparable octane number to gasoline, higher energy density, lower Reid vapour pressure and is less water miscible and less hygroscopic compared to bioethanol [6].

Biobutanol from renewable substrates including non-food lignocellulosic biomass are more suitable as fermentation feedstock as it does not compete with food crops demand [5]. In Malaysia, oil palm empty fruit bunch (OPEFB) fibres are the most abundant biomass produced at the palm oil mill with an annual production of 69,000 dry tonnes [7]. The OPEFB fibres are cheap substrate, which is suitable to be used as a fermentation feedstock for biobutanol production. Besides, utilisation of OPEFB fibres for biobutanol production could solve the inefficient OPEFB fibres disposal management which currently practice is by dumping at the mill or used as mulching agent at the plantation [8]. OPEFB fibres contain 17–26% of lignin that bind the cellulose and hemicellulose tightly. Therefore, a pretreatment step is essential to loosen the lignin component and expose the cellulose and hemicellulose component to be hydrolysed by cellulase into fermentable sugars. The pretreatment processes that can be used are mechanical, chemical, physicochemical, biological or a combination of them [9,10]. Chemical pretreatment using 2% NaOH has been found to be a suitable pretreatment for OPEFB fibres that could generate 32 g/L of sugar concentration with a yield of above 70% [11,12]. In addition, it can also reduce lignin to 12% and increase holocellulose content up to 80% (cellulose 54–59% and hemicellulose 22–28%) [13]. The holocellulose can be hydrolysed into fermentable sugars that consisted of pentoses (xylose and arabinose) and hexoses (glucose, galactose, and mannose) and that can be consumed by *Clostridia* to produce biobutanol [14].

Generally, there are four steps involved in a conversion of biobutanol from OPEFB fibres, which are; (1) pretreatment, (2) enzymatic saccharification, (3) ABE fermentation and (4) biobutanol recovery and purification [15]. Normally, steps (2) and (3) are conducted separately and known as separate hydrolysis and fermentation (SHF). In recent studies, these two steps are combined by incorporating the cellulase, OPEFB fibres and *Clostridia* species within a single reaction vessel and this process are known as simultaneous saccharification and fermentation (SSF). SSF can reduce the number of steps, process duration and equipment as compared to SHF, which could save the operational cost [16]. Another advantages of SSF are it can reduce the inhibitory effect of glucose to β-glucosidase as the sugars are consumed by cells as soon as it is produced [17]. Besides, biobutanol yield from SSF is also higher than SHF [15]. However, the main challenge of the SSF is the different operating conditions of enzymatic saccharification and ABE fermentation, which can affect SSF performance [18]. The optimum operating temperature for saccharification is in the range of 40–50 °C while ABE fermentation is conducted at 30–37 °C [19]. Besides, initial pH facilitates the activity of cellulase and controls the shifting of the pathway in ABE fermentation [20,21]. The most suitable cellulase loading and substrate concentrations in SSF are not yet determined, and thus the SSF process was reported to generate low sugar production in the system [16]. Therefore, an extensive study by conducting one factor at a time (OFAT) and response surface methodology (RSM) were carried out in order to optimise the SSF conditions and statistically analyses the relationship between the factors affecting the biobutanol production.

## 2. Materials and Methods 

### 2.1. Pretreatment of Oil Palm Empty Fruit Bunch 

Pressed and shredded OPEFB fibres were obtained from Seri Ulu Langat Palm Oil Mill Dengkil, Selangor, Malaysia. The OPEFB fibres were soaked in commercial detergent (50 mL/kg of OPEFB) overnight and washed with tap water to remove residual oil and dust as well as to prevent fungal contamination developed on the OPEFB fibre during storage. The OPEFB fibres were oven dried at 60 °C for 24 h before grinding using a hammer mill (Sima, Malaysia) to an average size of 3–5 mm. The 5% (*w*/*v*) of OPEFB fibres were soaked with 2% (*w*/*v*) NaOH for 4 h and autoclaved at 121 °C for 5 min [22]. The pretreated OPEFB fibres were washed with tap water to discard liquid containing degradation products and remaining NaOH until an approximately neutral pH of 7 was reached. The pretreated OPEFB fibres were oven dried at 60 °C and stored at room temperature prior to use. The cellulose, hemicellulose and lignin content were determined using the gravimetric method [23]. The lignocellulosic composition of the untreated OPEFB fibres were 43.51%, 34.26%, and 31.47%, while the alkaline pretreated OPEFB fibres consisted of 58.86%, 25.91% and 13.13%, of cellulose, hemicellulose and lignin, respectively.

### 2.2. Preparation of Inoculum

*Clostridium acetobutylicum* ATCC 824 employed in this study was purchased from American Type Culture Collection (ATCC, Manassas, VA, USA). The inoculum was prepared by transferring 1 mL of the *C. acetobutylicum* ATCC 824 stock culture into 99 mL of commercial Reinforced Clostridial Medium (RCM). The culture was incubated at 37 °C for 48 h in a static condition prior to inoculation into SSF [16]. Optical density (OD) of the bacterial inoculum was measured using spectrophotometer (Genesys 20, Thermo Scientific, Waltham, MA, USA) at 620 nm.

### 2.3. Medium Preparation and Fermentation

The SSF process was conducted using Acremonium cellulase (derived from *Acremonium cellulolyticus*, Meiji Seika Co., Tokyo, Japan), cellulase loading of FPU/g-substrate and 0.05 M of acetate buffer, pH 5.5. The cellulase activity of the Acremonium cellulase is 45 FPU/mL. The SSF working volume was conducted at 100 mL in 125-mL serum bottle.

SSF components were prepared by autoclaving 5% (*w*/*v*) pretreated OPEFB fibres at 121 °C for 15 min. The fermentation medium of SSF consisted of 12 g/L of yeast extract stock solution, 1 M and 0.05 M of acetate buffer stock solution and distilled water (for dilution purpose) was autoclaved at 121 °C for 15 min. The P2 medium, which comprised of the buffer, vitamins and mineral solutions were filter sterilised using 0.22 μm of nylon-driven filter [24]. Acremonium cellulase solution (25 FPU/mL) was prepared by dissolving the cellulase in 0.05 M acetate buffer (pH 5.5). 

SSF was conducted by adding 5 mL of 1 M of acetate buffer (pH 5.5), 26 mL of distilled water, 2 mL of each P2 medium components and 50 mL of 12 g/L of yeast extract into the serum bottle. A 3 ml of cellulase solution stock (filter sterilised) and 10% (*v*/*v*) of inoculum were added into the fermentation flask to initiate the SSF process. The experimental control, saccharification only (SO), had the same medium composition as SSF but without the introduction of the inoculum to exploit the sugars released from the OPEFB fibres. The SSF was carried out at 37 °C for 120 h with 150 rpm shaking speed. A 3 mL of the samples were withdrawn and was kept at −20 °C prior to analyses [16].

### 2.4. One Factor at a Time (OFAT)

The study on the effect of temperature (30–50 °C), initial pH (4.5–7.0), cellulase loading (5–30 FPU/g-substrate) and substrate concentration (1–7% *w*/*v*) were conducted using the OFAT approach. These factors were conducted sequentially by varying the investigated factor while keeping the others at constant. The significant effect of each the factor was analysed using Tukey’s test by Statistical Analysis Software (SAS) version 9.4, SAS Institute Inc., Cary, NC, USA. The statistical significance was verified considering *p* < 0.05.

### 2.5. Central Composite Design (CCD)

The parameter optimisation of SSF was conducted using central composite design (CCD) in Design Expert Software 7.0.0 (Stat-Ease, Inc., Minneapolis, MN, USA). Four independent factors were selected based on results from OFAT which are temperature (25–45 °C), initial pH (3.5–7.5), cellulase loading (5–25 FPU/g-substrates) and substrate concentration (1–9% *w*/*v*) with biobutanol yield as a response. Inoculum concentration was fixed at 15% (*v*/*v*). The CCD experimental matrix was designed for 30 experimental runs conducted in triplicate. The quadratic effects and interactions between significant factors towards biobutanol yield were calculated and the statistical significance of the model was verified using analysis of variance (ANOVA) with a significant *p*-value less than 0.05. A quadratic polynomial equation was used to describe the relationship of the factors towards the response and the model behaviour was explained by the following second order polynomial equation. 3D surface plots were used to show the effects of the investigated factors upon the biobutanol yield and to detect the optimal levels. Further validation was conducted using the predicted optimum conditions suggested by the model.

### 2.6. Analytical Methods

A filter paper unit (FPU) assay was conducted to measure the cellulase activity of Acremonium cellulase [25]. The reducing sugar concentration was determined using dinitrosalicylic acid (DNS) [26]. The pH was measured using a pH meter (Mettler Toledo, Columbus, OH, USA). The ABE and organic acids concentrations were determined using gas chromatograph GC-17A (Shimadzu, Kyoto, Japan) equipped with column BP20 and flame ionisation detector (FID) [16]. The cell concentration was determined based on the OD measured at 620 nm using a spectrophotometer (Genesys 20, Thermo Scientific, Waltham, MA, USA) [16]. The error bar (±) refers to the value of standard deviation. Biobutanol yield is defined by the mass of biobutanol produced (g) per mass of total sugars (g) (g of butanol/g of sugars). The overall conversion of substrate into biobutanol was defined in biobutanol yield of the mass of biobutanol produced (g) per mass of substrate concentration (g) (g of butanol/g of substrate). Biobutanol productivity was calculated as biobutanol produced in g/L divided by the fermentation time and expressed as g/L/h. The hydrolysis yield was calculated as in the following Equation 1:

(1) $$Hydrolysis\;yield=\frac{\left[reducing\;sugar\;\left(g/L\right)\times \;0.9\;\times \;100\right]}{\left[substrate\;concentration\;\left(g/L\right)\times \left(cellulose\;\%+hemicellulose\;\%\right)\right]},$$

## 3. Results and Discussion

### 3.1. Effect of Temperature

The highest biobutanol concentration and yield of 1.96 g/L and 0.08 g/g, respectively, were obtained at 35 °C and was statistically significant (*p* < 0.05) as compared with other temperatures (Table 1). An optimum biobutanol production was also obtained at 35 °C when *C. acetobutylicum* CICC 8008 was grown in corn straw hydrolysate [27]. Although a higher sugar concentration was produced at 37 °C, the cells prefer to produce more biobutanol at 35 °C. This was due to the reducing sugars produced in the system were consumed for the synthesis of the entire pool of fermentation products including acetone, ethanol, acetic acid, butyric acid, carbon dioxide, hydrogen and for cell formation [28]. 

Biobutanol production did not increase when the temperature was set higher than 40 °C or lower than 35 °C. This study showed that at the temperature higher than 35 °C, the sugars consumption decreased by more than 65%. *C. acetobutylicum* loses the ability to convert sugars into acids and reassimilates the acids into biobutanol at a temperature above 37 °C [29]. The sugars production decreased when the temperature was set above 40 °C and this might be due to the complex medium composition of SSF used for the SO, which inhibits the cellulase. It should be noted that a normal saccharification medium only consists of buffer solution and sodium azide [30]. Besides, the study also showed that SSF relieves the inhibition of sugar on cellulase as it is being consumed by the bacteria and released sugars higher than SO. Moreover, higher acetic and butyric acid were produced at temperature 30 °C as compared with other temperatures. High concentration of acids is toxic to the cells, causing the acid crash and cease the cell metabolism [31]. It was reported that the amount of acid should not be more than 13 g/L to avoid acid crash [13]. 

### 3.2. Effect of Initial pH

The initial pH significantly affected the biobutanol production in SSF. High biobutanol concentration and yield of 1.96 g/L and 0.08 g/g, respectively were obtained at pH 5.5 (Table 2). The initial pH 5.5 was found as the best metabolic state for biobutanol production as it does not create an initial inhibitory acidic condition for cell growth [32]. Therefore, a constant difference of pH values between internal cells fluid and external pH medium can be maintained [33]. This is important for the metabolism and growth of cells associated with the shifting from acidogenesis into solventogenesis which is crucial for acids and subsequent biobutanol production [21]. 

A total of 11.43 g/L of acids (7.43 g/L acetic and 4 g/L butyric) were observed in SSF at pH 5.5, which is higher than other pH conditions. In ABE fermentation by *Clostridia*, acids are produced during acidogenic phase before reassimilated into the cells for solvents production [34]. Therefore, it is important to produce a suitable amount of acids in the system to trigger the cells to produce biobutanol [20]. Initial pH lower than 5.5 is unfavourable for biobutanol production, where only 0.25 g/L of biobutanol concentration was obtained at pH 4.5. It should be noted that pH 4.5 was reported as a suitable cellulase working range for enzymatic saccharification of lignocellulosic biomass into fermentable sugars [8,35]. Thus, the highest amounts of fermentable sugars were observed at pH 4.5 as compared with other initial pH conditions. Besides, the results also showed that pH approaching to 7 is not suitable for biobutanol production in the SSF process. High pH value ceased the cells growth, impaired the acidogenic pathway and further decreased the solventogenic phase, which resulted in a low biobutanol production in the system [36].

### 3.3. Effect of Cellulase Loading

Optimal cellulase loading is very important in bioconversion of lignocellulosic biomass into biobutanol. This is because of the use of cellulase accounts for over 20% of total production cost for SSF process [37]. Therefore, it is essential to avoid excess cellulase loading in the system. In this study, the highest biobutanol production was recorded at cellulase loading of 10 FPU/g-substrate although the amount of sugar produced in SO is lower as compared with other cellulase loadings (Table 3). SSF with cellulase loading from 20–30 FPU/g-substrate decreased biobutanol production by 50%. Low sugar consumption was observed in SSF with high cellulase loading, which indicates an unfavourable condition for microbial growth. The cells might not be able to grow and produce biobutanol due to the stress caused by high cellulase loading [38]. Further increment of cellulase loading may lead to inhibitory action due to the high sugar concentration accumulated in the system [39]. An increasing amount of sugars estimated by the SO probably resulted in consumption of *Clostridia* towards producing acids that underwent low conversion of biobutanol in SSF. The result showed a significant reduction in biobutanol yield from 0.07 g/g to 0 g/L and displayed similar total acids production of less than 9 g/L. 

### 3.4. Effect of Substrate Concentration

A suitable amount of substrate concentration is very important in SSF. SSF employs solid substrate that needs to be saccharified simultaneously with the fermentation process. An excess amount of solid substrate in the system can cause inefficient mixing and subsequently reduced the saccharification performance [40]. In this study, 1–7% (*w*/*v*) of pretreated OPEFB fibres were applied to SSF. Results showed that 5% substrate concentration produced the highest biobutanol concentration of 2.47 g/L with biobutanol yield of 0.087 g/g (Table 4). The estimated amount of sugar produced in the system (SO) was 18.73 g/L, which was observed as a suitable sugar concentration for cells to produce biobutanol.

Besides, OPEFB fibres cannot be degraded directly by *C. acetobutylicum* ATCC 824 as the cells do not produce cellulase extracellularly [19]. Therefore, suitable cellulase loading associated with substrate concentration should be identified to produce a sufficient amount of fermentable sugars for microbial consumption. Low sugar concentration at 1% (*w*/*v*) substrate concentration produced the lowest biobutanol concentration of 0.50 g/L. Low substrate concentration tends to produce low fermentation products according to the law of mass action [41]. Low biobutanol was produced at a maximum substrate concentration of 7% (*w*/*v*), possibly due to the acid crash phenomenon as higher acids were produced instead of biobutanol. Besides, an inefficient mixing process was observed in substrate concentration of 7% (*w*/*v*) in the first 24 h of SSF. The mixture becomes fluid after 24 h and the saccharification was improved. However, the sugar produced was consumed by the cells to produce acids and the cells’ metabolism ceased before they enter the solventogenic phase. The biobutanol production was improved 1.42 fold with 29.55% increment which resulted in maximum biobutanol concentration of 2.47 g/L and biobutanol yield 0.10 g/g.

### 3.5. Optimisation of Biobutanol Production

Central composite design (CCD) was implemented to optimise four factors (temperature, initial pH, cellulase loading, and substrate concentration) as well as to develop the correlation between the four factors that affect the biobutanol yield. The coded and real values of the factors were ttemperature (A), initial pH (B), cellulase loading (C), and substrate concentration (D) (Table S1). The CCD experimental matrix with 30 experimental runs (Table S2) were randomised for statistical analysis in order to minimize the effects of unexplained variability in the observed responses [36]. In this experimental matrix, six identical runs of the centre point were evaluated to check the experimental variability for an internal estimate of the CCD error [42]. From this experiment, the biobutanol yield recorded was in the range of 0 to 0.16 g/g (Table S2). To describe the relationship between the significant factors and the biobutanol yield in terms of decoded values, the CCD data were fitted to a second-order quadratic polynomial as in Equation (2), by applying multiple regression analysis.

(2) $$\;Y\;=\;0.14\;+\;4.542\;\times \;10^{-3}\;\times \;A\;+\;6.958\;\times \;10^{-3}\;\times \;B\;+\;5.417\;\times \;10^{-4}\;\times \;C+1.208\;\times \;10^{-3}\;\times \;D\;-\;3.188\;\times \;10^{-3}\;\;\;\times \;A\;\times \;B\;-\;5.438\;\times \;10^{-3}\;\times \;A\;\times \;C\;-\;6.313\;\times \;10^{-3}\;\times \;A\;\times \;D\;-\;7.563\times \;10^{-3}\;\times \;B\;\times \;C\;+\;3.813\;\times \;10^{-3}\;\;\times \;B\;\times \;D\;-\;4.188\;\times \;10^{-3}\;\times \;C\;\times \;D\;-\;0.029\;\times \;A^{2}\;-\;0.030\;\times \;B^{2}\;-\;0.026\;\times \;C^{2}\;-\;0.031\;\times \;D^{2}$$

*Y* is the experimental response for biobutanol yield and the function A, B, C, D are the coded values of temperature, initial pH, cellulase loading and substrate concentration, respectively. 

The experimental results obtained were fitted to a quadratic polynomial and generate second-order model (Table S3). The statistical significance of the second-order quadratic polynomial equation for the experimental data was analysed using ANOVA as shown in Table 5. The overall quadratic model showed that it is significant (*p* < 0.05) while the lack of fit was non-significant indicating the experimental results obtained well fitted with the model. The significance of the results is judged by its *p*-value closer to 0.

**Figure 1:**
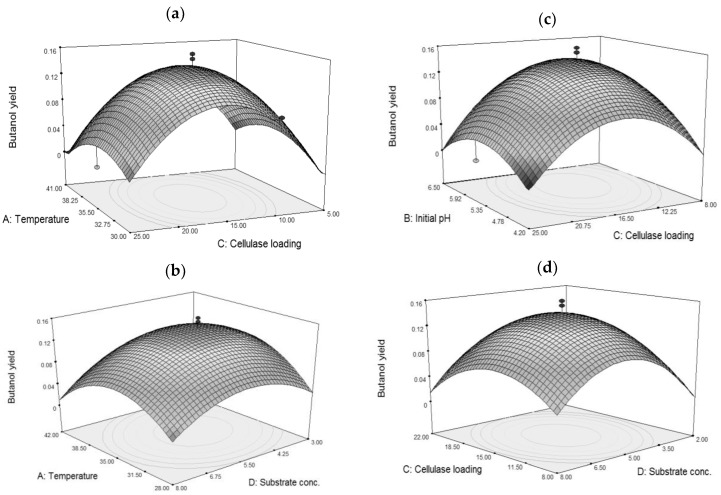
3D-surface graphs for a model for biobutanol yield at the optimum point: (**a**) interaction between temperature and cellulase loading, (**b**) interaction of temperature and substrate concentration, (**c**) interaction of initial pH with cellulase loading, (**d**) interaction of cellulase loading with substrate concentration.

The 3D response surface plots were shown in Figure 1, which depicted the interactions between two factors keeping the other factor at zero level for biobutanol yield. In the design boundary of the 3D plots, each response surface plot had a clear peak and the corresponding contour plot had a clear highest point, which defined that the maximum biobutanol yield could be achieved within the design boundary and not in the extreme plot. The biobutanol yield increased with increasing temperature, initial pH, cellulase loading and substrate concentration up to the optimal conditions. Declination of biobutanol yield was observed when factor levels increased more than optimal conditions.

Four different independent factors (temperature, initial pH, cellulase loading and substrate concentration) were statistically found to have little effect on biobutanol yield; however, these factors showed biological importance when dealing with the nature of the SSF process. Temperature played a pivotal role in SSF by affecting two main components which are cellulase and *C. acetobutylicum* ATCC 824. These two components have different optimum operating temperatures, which subsequently influenced hydrolysis of OPEFB fibres and biobutanol production. The commercial Acremonium cellulase used in this study was produced by *Acremonium cellulolyticus* fungi that capable to hydrolyse at the temperature range of 30−60 °C [43]. However, the optimum temperature for SSF was observed at 35 °C, in order to compromise with the growth of *C. acetobutylicum* ATCC 824 for maximum biobutanol yield. 

Understanding the relationship of initial pH in SSF between Acremonium cellulase and *C. acetobutylicum* ATCC 824 was crucial as initial pH is the key factor in biobutanol production. During the first 24 h of SSF, *C. acetobutylicum* ATCC 824 metabolism is required to reduce the pH from 5.5 to 4.95 (a breakpoint value) by producing organic acids. This pH value will then increase back after the transition phase to solventogenesis is successful. This pH reduction showed no restriction on the enzymatic hydrolysis activity as the Acremonium cellulase is capable of working within pH 4 to 5 [43]. 

The effect of cellulase loading was statistically shown as the most insignificant factor affecting biobutanol yield in the SSF process. However, cellulase loading contributes as the most important factor for the whole operational cost. The cost of cellulase needs to be reduced in order to make the SSF process economically viable [37]. This cost reduction can be achieved by implementing low cellulase loading, as the high price of cellulase acts as a barrier for conversion of lignocellulosic biomass [44]. In this study, cellulase loading of 10 FPU/g-substrate led to significantly higher biobutanol production; meanwhile, in CCD by RSM, the cellulase loading corresponding to the maximum biobutanol yield was 15 FPU/g-substrate. Besides, Acremonium cellulase also possesses hemicellulases such as xylanase which can increased the yields of sugar production and decreased the loading of cellulase required for enzymatic saccharification [45]. 

The substrate concentration provides fermentable sugars (enzymatic hydrolysis) as a substrate for *C. acetobutylicum* ATCC 824 to produce biobutanol. In SSF, the OPEFB fibres in the serum bottles were utilised directly for biobutanol production. Several potential assessment steps to conduct an SSF process may include characteristic, type and size of biomass and the shape of the vessel used. When the substrate concentration is increased, the SSF tends to generate a low biobutanol yield. As observed in the high substrate load (9%) in SSF, the substrate was saturated up to the top of serum bottles and experienced limited headspace volume, therefore affecting the stirring and mixing process to become slower at the beginning of the fermentation. This resulted in mass and heat transfer problems, which eventually produced low biobutanol yield.

### 3.6. Validation of Biobutanol Optimisation

**Figure 2:**
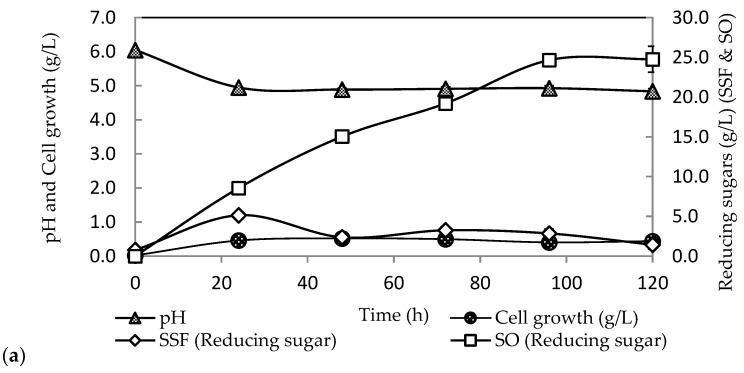

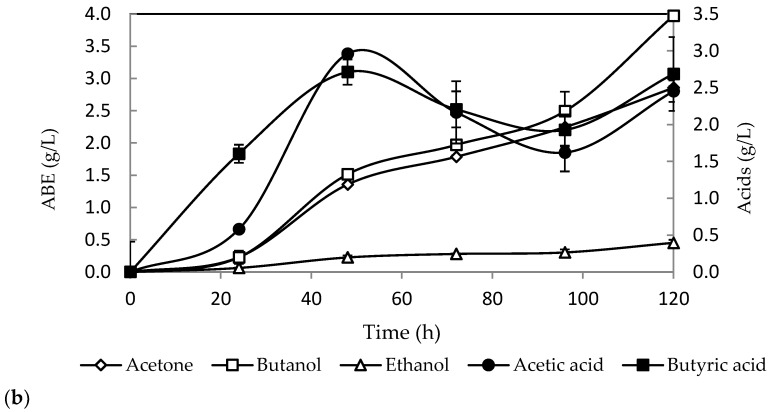
Simultaneous saccharification and fermentation (SSF) for biobutanol using oil palm empty fruit bunch by *Clostridium acetobutylicum* ATCC 824 at 35 °C, initial pH 5.5, cellulase loading of 15 FPU/g-substrate and 5% (*w*/*v*) oil palm empty fruit bunch (OPEFB) fibres. (**a**) The time courses for reducing sugar in SSF and SO and pH in the SSF system and (**b**) The ABE and acids concentration obtained from SSF process.

Validation of biobutanol optimisation was performed under optimal conditions obtained from the CCD experiment to validate the accuracy of RSM prediction. From this validation, Figure 2a showed that the pH in the SSF system dropped rapidly within the first 24 h, suggesting that the culture produced acids at a rapid rate. After 40 h, it remained at a constant level until 120 h of fermentation. Figure 2b shows acetic and butyric acid productions were obtained at low concentrations of 2.45 g/L and 2.69 g/L, respectively. Most of the acids were successfully reassimilated and converted into ABE. It should be noted that, as compared to OFAT, a higher amount of acids produced in the system might be due to unoptimised fermentation conditions, making it unfavourable for cells to produce biobutanol. This study showed that the optimum conditions of SSF improved the conversion of acids into solvents, especially biobutanol.

The sugar profile in SSF had a fluctuated pattern whereby the sugars were rapidly consumed by cells at 24 to 48 h. The sugars produced in the system were consumed for acetic and butyric acids formation that can be observed after 24 to 48 h of fermentation. The acetone and ethanol were seen to increase alongside with biobutanol with 2.14-fold increment. The total ABE concentration reached up to 7.38 g/L. The residual sugars in SSF were 1.43 g/L, which is less than the residual sugar in OFAT. Under optimum conditions, *C. acetobutylicum* ATCC 824 completely metabolised almost 94% of the fermentable sugars released by the hydrolysis of pretreated OPEFB fibres in SSF. The hydrolysis yield of OPEFB fibres by Acremonium cellulase in the SSF was 53%, which released about 25 g/L of fermentable sugars.

The validation of the optimised fermentation parameters produced 0.16 g/g of biobutanol yield with 3.97 g/L of biobutanol concentration. The predicted biobutanol yield generated by Equation (1) from CCD using optimised conditions was 0.14 g/g, accounting approximately for 12.5% error as compared to the experimental biobutanol yield. This result signified the increment of biobutanol production at the optimum conditions during the validation trial. It is suggested that the RSM approach was effective in optimising the operational conditions for biobutanol production through SSF.

The maximum biobutanol was obtained at 120 h; therefore, biobutanol productivity remained at 0.03 g/L/h as compared to OFAT. The transition point from acidogenesis into solventogenesis took almost 48 h. This transition was similar to SSF that performed the intensification of sugar production at high solid content [46]. A shorter fermentation time was achieved with an earlier transition point from acids to solvents production. However, this current study is lacking in promoting high initial sugars or intensification of sugars in the SSF process which probably could shift the transition to an earlier state thus producing biobutanol at a faster rate. The productivity of the SSF plays a critical role for a larger scale. Low biobutanol productivity is unbeneficial as it takes a longer time for operation, which adds more cost and directly affects the process economics and commercial feasibility of SSF [38]. However, biobutanol concentration obtained can be of higher importance compared to productivity for industrial applications as product recovery efficiency is very much affected by its concentration [47]. 

A comparison of biobutanol production from various substrates through the SSF process is shown in Table 6. This optimisation study significantly increased the biobutanol yield and showed higher fermentability at optimum SSF operating conditions. In this study, the biobutanol yield (0.16 g/g) was comparable to the study that uses two types of the substrate which are avicel (0.16 g/g) and de-ashed paper mill sludge (0.14 g/g) [48]. It was suggested that the optimum conditions of SSF used in this study could be adapted to produce biobutanol from pretreated OPEFB fibres. The highest biobutanol yield (0.31 g/g) from the SSF process was obtained using steam-exploded wood chips [17]. In comparison for an overall conversion of substrates to biobutanol, this study produced biobutanol yield (0.08 g/g-substrate), which was comparable to SSF using corncob that yielded biobutanol of 0.05 g/g-substrate [49]. The overall biobutanol conversion yield (from the raw substrate to biobutanol) is beneficial for an estimation capability of SSF for larger scale. An overall summary of the productivity from this study is 0.03 g/L/h, which was comparable with other studies (<0.08 g/L/h).

Although biobutanol represents a superior and renewable liquid fuel, there are several major drawbacks involved in processing biobutanol from low-cost OPEFB fibres through the SSF process. Since this study was only conducted at laboratory scale, limitations or challenges may occur for a future scale-up process. This involves a series of processing steps, which are washing with detergent, drying, size reduction, pretreatment, washing and drying. There is also a huge amount of wastewater released due to the processing of OPEFB fibres. A sophisticated washing system is needed to reduce the amount of wastewater released. Besides, the SSF process also has a limitation, whereby the saccharification process from OPEFB fibres produced a low amount of sugars for biobutanol production. Further research is needed to tackle these issues by increasing substrate concentration without limiting stirring process or mass transfer and with the aim of increasing sugars released from the biomass in the overall process, as well as providing sugars at the initial stages of the SSF process. The temperature is also an important criterion in SSF, where it significantly affects sugars production and must also satisfy *Clostridia* growth responsible for biobutanol production. A method of prehydrolysis of the SSF process or integration of a thermophilic strain known as the consolidated bioprocessing technology should be conducted to improve these issues. These drawbacks will subsequently result in inefficient biobutanol recovery and purification, thus making the whole process unviable for a large-scale production. However, there is a study showing that integration of SSF with recovery (SSFR) is possible and can produce higher ABE concentration, yield and productivity [50]. A study conducted a recovery of ABE from oil palm biomass with biobutanol concentration of 3.5 g/L, which is similar to this study [51]. Nevertheless, with the integration of new bacterial strains and with highly sophisticated pretreatment, advancement of SSF and downstream process, biobutanol production through SSF process may possibly be introduced at an industrial scale.

## 4. Conclusions

The present study showed that OPEFB fibres could serve as a potential substrate for biobutanol production from SSF by *Clostridium acetobutylicum* ATCC 824. The optimised fermentation conditions of SSF led to a significant maximum biobutanol concentration of 3.97 g/L with a biobutanol yield of 0.16 g/g, equivalent to a 55.95% increment (2.14-fold) with almost 94% of sugars consumed by *Clostridium acetobutylicum* ATCC 824. Temperature, initial pH, cellulase loading and substrate concentration showed biological importance towards the SSF process and were needed in an optimal amount to obtain high biobutanol production. The model and optimisation design obtained in this study have helped to explore the interaction effects of those factors towards biobutanol production and improve this as well.

## Figures and Tables

**Table 1:** Effect of temperature (30–50 °C) on biobutanol production at initial pH 5.5, cellulase loading of 15 FPU/g-substrate and 5% (*w*/*v*) substrate on simultaneous saccharification and fermentation by *Clostridium acetobutylicum * ATCC 824.

Temperature (°C)	30	35	37	40	45	50
Solvents and acids production						
Biobutanol (g/L)	1.53 ± 0.15 ^a^	1.96 ± 0.04 ^a^	1.74 ± 0.06 ^a^	1.55 ± 0.07 ^a^	0.08 ± 0.02 ^b^	0.07 ± 0.003 ^b^
Acetic acid (g/L)	10.97 ± 0.86	7.43 ± 0.50	7.37 ± 0.11	6.27 ± 0.05	6.72 ± 0.33	6.35 ± 0.25
Butyric acid (g/L)	6.86 ± 0.69	4.00 ± 0.45	3.67 ± 0.23	3.32 ± 0.19	2.65 ± 0.02	2.80 ± 0.47
Total acetone-butanol-ethanol (ABE) concentration (g/L)	2.01	2.62	2.74	2.14	0.08	0.07
Total ABE yield (g ABE/g sugar)	0.08	0.10	0.11	0.08	0.003	0.003
Biobutanol yield (g butanol/g sugar)	0.06	0.08	0.07	0.06	0.003	0.003
Biobutanol productivity (g/L/h)	0.015	0.02	0.02	0.016	0.0008	0.0007
Final pH	4.81 ± 0.08	4.92 ± 0.11	4.82 ± 0.09	4.85 ± 0.03	4.45 ± 0.09	4.95 ± 0.38
Reducing sugars (g/L)						
SSF	3.84 ± 0.18	4.54 ± 0.03	8.38 ± 0.01	13.00 ± 0.79	19.99 ± 0.17	22.53 ± 0.22
SO	10.90 ± 0.93	19.55 ± 0.39	25.61 ± 0.27	23.49 ± 0.00	21.75 ± 0.26	16.38 ± 0.06

Data obtained at 96 h of fermentation; Data in the same row with different superscript letters are significantly different (*p* < 0.05); SSF—simultaneous saccharification and fermentation; SO—saccharification only; ^a, b^ Row means with different superscripts differ significantly (*p* < 0.05) within parameter means.

**Table 2:** Effect of initial pH (4.5–7.0) on biobutanol production at 35 °C, cellulase loading of 15 FPU/g-substrate and 5% (*w*/*v*) substrate on simultaneous saccharification and fermentation by *Clostridium acetobutylicum* ATCC 824.

Initial PH	4.5	5.0	5.5	6.0	6.5	7.0
Solvents and acids production						
Biobutanol (g/L)	0.25 ± 0.04 ^c^	1.06 ± 0.18 ^b^	1.96 ± 0.04 ^a^	1.22 ± 0.02 ^c^	0.64 ± 0.20 ^c^	0.57 ± 0.40 ^c^
Acetic acid (g/L)	3.91 ± 0.05	4.11 ± 0.00	7.43 ± 0.50	3.34 ± 0.17	3.34 ± 0.42	3.39 ± 0.00
Butyric acid (g/L)	4.18 ± 0.25	2.87 ± 0.97	4.00 ± 0.45	2.40 ± 0.27	2.65 ± 0.29	2.75 ± 0.00
Total ABE concentration (g/L)	0.46	1.52	2.62	1.77	1.05	0.90
Total ABE yield (g ABE/g sugar)	0.02	0.06	0.10	0.07	0.04	0.04
Biobutanol yield (g butanol/g sugar)	0.01	0.04	0.08	0.05	0.03	0.02
Biobutanol productivity (g/L/h)	0.002	0.02	0.02	0.01	0.006	0.006
Final pH	4.75 ± 0.29	4.60 ± 0.14	4.92 ± 0.11	4.60 ± 0.10	4.61 ± 0.14	4.69 ± 0.15
Reducing sugars (g/L)						
SSF	12.46 ± 0.28	7.65 ± 0.14	4.54 ± 0.03	8.52 ± 0.00	9.49 ± 0.04	8.63 ± 0.03
SO	24.80 ± 0.98	22.67 ± 0.17	19.55 ± 0.39	17.66 ± 0.31	18.55 ± 1.33	19.30 ± 0.17

Data obtained at 96 h of fermentation; Data in the same row with different superscript letters are significantly different (*p* < 0.05); SSF—simultaneous saccharification and fermentation; SO—saccharification only; ^a, b, c^ Row means with different superscripts differ significantly (*p* < 0.05) within parameter means.

**Table 3:** Effect of cellulase loading (5–30 FPU/g-substrate) on biobutanol production at 35 °C, initial pH 5.5 and 5% (*w*/*v*) substrate on simultaneous saccharification and fermentation by *Clostridium acetobutylicum* ATCC 824.

Cellulase Loading (FPU/g-Substrate)	5	10	15	20	25	30
Solvents and acids production						
Biobutanol (g/L)	0.70 ± 0.10 ^b^	2.31 ± 0.30 ^a^	1.92 ± 0.05 ^b^	1.64 ± 0.45 ^b^	0.84 ± 0.25 ^c^	0.03 ± 0.00 ^c^
Acetic acid (g/L)	5.38 ± 0.44	4.75 ± 0.09	6.26 ± 0.27	4.38 ± 0.09	3.81 ± 0.84	5.97 ± 0.64
Butyric acid (g/L)	4.62 ± 0.30	3.53 ± 0.20	4.05 ± 0.36	4.35 ± 0.31	5.34 ± 0.42	3.74 ± 0.32
Total ABE concentration (g/L)	1.26	2.80	2.73	2.85	1.61	0.12
Total ABE yield (g ABE/g sugar)	0.05	0.11	0.11	0.11	0.063	0.005
Biobutanol yield (g butanol/g sugar)	0.03	0.09	0.07	0.07	0.033	0.001
Biobutanol productivity (g/L/h)	0.007	0.024	0.02	0.017	0.009	0.0003
Final pH	4.69 ± 0.08	4.78 ± 0.02	4.85 ± 0.08	4.72 ± 0.06	4.58 ± 0.01	4.55 ± 0.15
Reducing sugars (g/L)						
SSF	0.62 ± 0.05	0.90 ± 0.00	5.91 ± 0.75	9.71 ± 0.13	11.63 ± 0.06	14.06 ± 0.83
SO	7.61 ± 0.79	11.51 ± 0.08	11.84 ± 0.40	17.91 ± 0.04	21.12 ± 0.95	22.68 ± 0.15

Data obtained at 96 h of fermentation; Data in the same row with different superscript letters are significantly different (*p* < 0.05); SSF—simultaneous saccharification and fermentation; SO—saccharification only; ^a, b, c^ Row means with different superscripts differ significantly (*p* < 0.05) within parameter means.

**Table 4:** Effect of substrate concentrations (1–7% *w*/*v*) on biobutanol production at a temperature of 35 °C, initial of pH 5.5 and cellulase loading of 10 FPU/g-substrate on simultaneous saccharification and fermentation by *Clostridium acetobutylicum* ATCC 824.

Substrate Concentration *w*/*v* (%)	1	3	5	7
Solvents and acids production				
Biobutanol (g/L)	0.50 ± 0.03 ^c^	0.95 ± 0.01 ^c^	2.47 ± 0.06 ^a^	1.79 ± 0.03 ^b^
Acetic acid (g/L)	3.58 ± 0.16	4.71 ± 0.61	3.52 ± 0.62	4.51 ± 0.32
Butyric acid (g/L)	2.54 ± 0.10	4.93 ± 0.13	4.36 ± 0.34	5.88 ± 0.41
Total ABE concentration (g/L)	0.54	1.29	4.34	3.48
Total ABE yield (g ABE/g sugar)	0.02	0.05	0.17	0.14
Biobutanol yield (g butanol/g sugar)	0.02	0.04	0.10	0.07
Biobutanol productivity (g/L/h)	0.01	0.01	0.03	0.02
Final pH	5.29 ± 0.10	4.82 ± 0.01	4.85 ± 0.04	4.77 ± 0.06
Reducing sugars (g/L)				
SSF	0.48 ± 0.05	0.35 ± 0.20	1.73 ± 0.12	8.65 ± 2.74
SO	1.29 ± 0.72	12.01 ± 0.19	18.73 ± 0.52	28.20 ± 0.44

Data obtained at 96 h of fermentation; Data in the same row with different superscript letters are significantly different (*p* < 0.05); SSF—simultaneous saccharification and fermentation; SO—saccharification only; ^a, b, c^ Row means with different superscripts differ significantly (*p* < 0.05) within parameter means.

**Table 5:** The analysis of variance (ANOVA) results for the response of biobutanol yield.

Source	Sum of Squares	df	MS	F Value	*p*-Value	
Model	0.06915	14	0.00494	5.62465	0.0010	Significant
A-Temperature	0.00050	1	0.00050	0.56371	0.4644	
B-Initial pH	0.00116	1	0.00116	1.32323	0.2680	
C-Cellulase loading	0.00001	1	0.00001	0.00802	0.9298	
D-Substrate conc.	0.00004	1	0.00004	0.03990	0.8444	
AC	0.00047	1	0.00047	0.53868	0.4743	
AD	0.00064	1	0.00064	0.72600	0.4076	
BC	0.00092	1	0.00092	1.04199	0.3235	
CD	0.00028	1	0.00028	0.31948	0.5803	
A2	0.02312	1	0.02312	26.32385	0.0001	
B2	0.02474	1	0.02474	28.16857	<0.0001	
C2	0.01841	1	0.01841	20.96173	0.0004	
D2	0.02599	1	0.02599	29.59310	<0.0001	
Residual	0.01317	15	0.00088	-	-	Not significant
Lack of Fit	0.01116	10	0.00112	2.76894	0.1362	
Pure Error	0.00201	5	0.00040	-	-	
Cor Total	0.08233	29	-	-	-	

R^2^ = 0.8399, Adj R^2^ = 0.6907.

**Table 6:** Comparison studies of biobutanol production through SSF process from various substrates.

Clostridium Strain	Temp. (°C)	Initial PH	Substrate Conc. (g/L)	Sugar Conc. (g/L)	Cellulase Loading	Biobutanol Conc. (g/L)	Biobutanol Yield		Biobutanol Productivity (g/L/h)	References
							(g/g) (Biobutanol/Sugar)	(g/g) (Biobutanol/Substrate)		
*C. acetobutylicum* NBRC 13948	37	5.0	50 g/L Wood chips	25	7.8 mg protein/g of substrate	7.77	0.31	0.16	0.05	[17]
*C. beijerinckii* TISTRI461	37	6.6	40 g/L Corncob	8	10 FPU/g-substrate	2.00	0.25	0.05	0.03	[49]
*C. acetobutylicum*	36	6.7	58 g/L Avicel	60	20 FPU/g of glucan	9.50	0.16	0.16	0.08	[48]
		6.7	74 g/L De-ashed paper mill sludge	71	10 FPU/g of glucan	9.70	0.14	0.13	0.08	
*C. acetobutylicum* ATCC 824	35	5.5	50 g/L OPEFB	25	15 FPU/g-substrate	3.97	0.16	0.08	0.03	This study

## Supplementary Materials

The following are available online at http://www.mdpi.com/1420-3049/23/8/1944/s1, Table S1: Coded values for each factor of the central composite design (CCD) for biobutanol production in SSF, Table S2: Experimental data of central composite design (CCD) for biobutanol yield, Table S3: The ANOVA for the second order model of central composite design (CCD) for biobutanol yield.
