# Supplementary material for: Optimisation of Simultaneous Saccharification and Fermentation (SSF) for Biobutanol Production Using Pretreated Oil Palm Empty Fruit Bunch
Source: Molecules. 2018 Aug 3;23(8):1944. doi: 10.3390/molecules23081944 (PMC6222772; doi:10.3390/molecules23081944)
Supplement: Supplementary file 1 [file molecules-23-01944-s001.pdf]

Supplementary Materials:

Table S1. Coded values for each factor of the central composite design (CCD) for biobutanol production in SSF.

Factors		Unit	Code values				
			-α	-1	0	+1	+α
A	Temperature	°C	25	30	35	40	45
B	Initial pH	-	3.5	4.5	5.5	6.5	7.5
C	Cellulase loading	FPU/g -substrate	5	10	15	20	25
D	Substrate concentration	% (w/v)	1	3	5	7	9

Table S2. Experimental data of central composite design (CCD) for biobutanol yield.

Run	A: Temperature	B: Initial pH	C: Cellulase loading	D: Substrate conc.	Response Y: Biobutanol yield (g butanol/g sugar)
1	40	4.5	20	7	0.025
2	30	6.5	20	3	0.024
3	30	4.5	20	3	0.001
4	40	6.5	10	3	0.049
5	30	4.5	20	7	0.022
6	30	4.5	10	3	0.001
7	35	7.5	15	5	0.032
8	40	4.5	20	3	0.093
9	45	5.5	15	5	0.004
10	40	4.5	10	7	0.021
11	30	6.5	10	3	0.011
12	40	6.5	20	7	0.008
13	35	5.5	15	5	0.153
14	35	5.5	15	5	0.129
15	40	6.5	10	7	0.076
16	35	5.5	5	5	0.058
17	40	4.5	10	3	0.000
18	30	6.5	10	7	0.001
19	40	6.5	20	3	0.033
20	35	5.5	25	5	0.008
21	35	5.5	15	5	0.160
22	35	5.5	15	1	0.009
23	35	5.5	15	5	0.163
24	35	5.5	15	5	0.120
25	35	3.5	15	5	0.001
26	35	5.5	15	5	0.120
27	25	5.5	15	5	0.037
28	35	5.5	15	9	0.018
29	30	6.5	20	7	0.068
30	30	4.5	10	7	0.002

Table S3. The ANOVA for the second order model of central composite design (CCD) for biobutanol yield

Source	Sum of square	Degree of freedom (df)	R^2	F value	*p*-value
Linear	1.699×10^{-3}	4	0.0206	0.13	0.9693
2FL	2.701×10^{-3}	6	0.0535	0.11	0.9942
Quadratic	0.065	4	0.8400	18.43	<0.0001
Cubic	0.011	8	0.9729	4.30	0.0351
